# A Rare Case of Metastatic Ampullary Adenocarcinoma Following the Whipple Procedure

**DOI:** 10.7759/cureus.46796

**Published:** 2023-10-10

**Authors:** Aboud Kaliounji, Haya Kaliounji, Michael A Farraj, Nischal Sharma, Samy I. McFarlane

**Affiliations:** 1 Internal Medicine, State University of New York (SUNY) Downstate Health Sciences University, Brooklyn, USA; 2 Internal Medicine, University of Colorado Anschutz Medical Campus, Aurora, USA; 3 Internal Medicine, Touro College of Osteopathic Medicine, Middletown, USA; 4 Internal Medicine, Nassau University Medical Center, East Meadow, USA; 5 Internal Medicine, St. George's University, True Blue, GRD

**Keywords:** whipple procedure, intestinal type, pancreatobiliary, periampullary carcinoma, ampullary carcinoma

## Abstract

Ampullary carcinoma is an extremely rare type of gastrointestinal cancer that originates at the ampulla of Vater, distal to the junction between the pancreatic duct and the common bile duct (CBD). There are three subtypes depending on the histological findings: pancreatobiliary, intestinal, and mixed subtype. Symptoms can mimic other pathologies related to biliary obstruction, such as jaundice, diarrhea, steatorrhea, and weight loss. In this report, we present a case of a 40-year-old male who presented with painless jaundice and dizziness. Magnetic resonance cholangiopancreatography (MRCP) showed choledocholithiasis and CBD dilatation. Endoscopic ultrasound showed a 24 x 14 mm ampulla mass. Subsequently, he underwent the Whipple procedure that revealed an intestinal-type periampullary adenocarcinoma characterized as stage III (T3bN2M0), with lymphovascular and perineural invasion. He was lost to follow-up but was later found to have metastatic pancreatic adenocarcinoma to the lung and liver. In this report, we also discuss the clinical presentation, pathogenesis, and current evidence-based therapeutic options in the management of this tumor, highlighting the importance of treatment choice depending on the tumor type.

## Introduction

Ampullary carcinomas originate at the ampulla of Vater, distal to the junction between the pancreatic duct and the common bile duct (CBD). They are rare malignancies, accounting for only 0.2% of gastrointestinal cancers and 7-20% of all periampullary cancers [1,2]. The overall incidence in developed countries is less than 0.5 per 100,000 based on the data from the international registries, with a slight dominance in males and a wide age range at diagnosis [1]. There are three main histologic subtypes of ampullary cancer: intestinal, which originates from the intestinal epithelium of the ampulla; pancreaticobiliary, which originates from the epithelium of the distal pancreatic and cystic ducts; and mixed type [2]. Unlike other periampullary cancers, ampullary cancers manifest symptoms early in the course with symptoms related to biliary obstruction, with the most common symptom being jaundice [3]. While the pancreaticobiliary type carries a worse prognosis, it is also the most prevalent type, followed by the intestinal type [4]. Overall, ampullary carcinomas have a better prognosis than other periampullary and pancreatic cancers, though therapy options and data are limited due to the rarity of the disease. In the early stages, ampullary cancers are typically treated surgically with the Whipple procedure. Though surgery may be curative at first, tumor recurrence is seen in around 50% of patients. Here we present a case of a 40-year-old male who was diagnosed with metastatic ampullary adenocarcinoma.

## Case presentation

**Figure 1:**
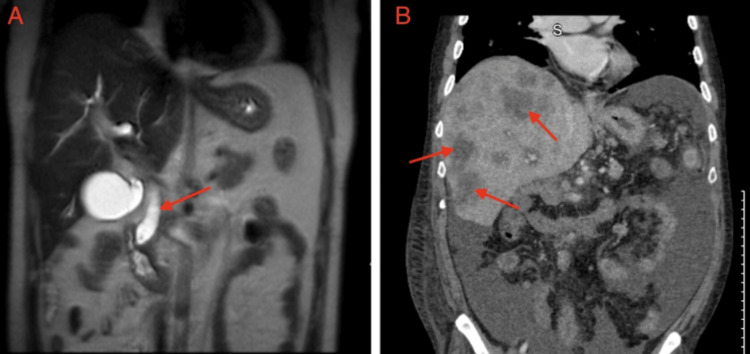
(A) MRCP showing common bile duct dilatation (red arrow). (B) Computed tomography of the abdomen and pelvis showing metastatic liver lesions (red arrows). MRCP, magnetic resonance cholangiopancreatography

A 40-year-old male with a history of alcohol use for more than 20 years presented to the emergency department with painless jaundice and dizziness for two weeks associated with occasional loose bowel movements. On arrival, his blood pressure was 144/86 mmHg, heart rate was 95 beats per minute, respiratory rate was 18 breaths/min, and temperature was 99.2°F. Physical examination was notable for upper abdominal tenderness, severe jaundice, and icterus. Laboratory tests were notable for a hemoglobin of 6.3 g/dL, as well as elevated alanine aminotransaminase and aspartate aminotransaminase (160 and 153 U/L, respectively), elevated alkaline phosphatase (513 U/L) and elevated total bilirubin (9.5 mg/dL), as shown in Table 1 along with other pertinent admission labs. He was subsequently transfused with 2 units of packed red blood cells with an appropriate response. General surgery was consulted to rule out acute cholecystitis, which did not recommend any surgical intervention. Magnetic resonance cholangiopancreatography (MRCP) showed small choledocholithiasis in the distal CBD, CBD dilatation, and no signs of acute cholangitis (Figure 1A). He was eventually transferred to another facility for endoscopic retrograde cholangiopancreatography (ERCP) and endoscopic ultrasound, which revealed a 24 mm x 14 mm ampulla mass and a dilated CBD with biopsy showing moderately differentiated adenocarcinoma without microsatellite instability. An ERCP was attempted but was unsuccessful due to the inability to visualize the ampulla for cannulation. Interventional radiology (IR) was then consulted for a percutaneous transhepatic cholangiogram and drain placement. Pre-procedure magnetic resonance imaging (MRI) showed a 1.9 x 1.4 cm periampullary mass and pancreatic tail lesion demonstrating rim enhancement, adding further suspicion for a solid pancreatic neoplasm. The patient underwent an exploratory laparotomy and pancreaticoduodenectomy, otherwise known as the Whipple procedure, one month after initial presentation. While the pathology of the pancreas tail lesion was benign, the periampullary mass pathology demonstrated a periampullary adenocarcinoma characterized as stage III (T3bN2M0) moderately differentiated intestinal type with lymphovascular invasion (LVI), 3 cm in size with 0.9-cm invasion into the pancreas. The pathology report further characterized the neoplasm as positive for both LVI and perineural invasion (PNI) and also found four out of nine lymph nodes to be positive for carcinoma. The patient followed up with oncology a month after surgery, where he was referred for a Mediport placement and radiation oncology evaluation.

**Table 1:** Laboratory results on admission BUN, blood urea nitrogen; ALT, alanine aminotransaminase; AST, aspartate aminotransaminase; WBC, white blood count; RBC, red blood cells; HGB, hemoglobin; HCT, hematocrit

	Lab Results	Reference
Anion gap	15	8-12 mEq/L
Sodium	125	135-145 mmol/L
Potassium	4.6	3.5-5.0 mmol/L
Chloride	91	98-108 mmol/L
CO_2_	19	22-32 mmol/L
BUN	8	6-26 mg/dL
Creatinine	0.41	0.7-1.2 mg/dL
Glucose	422	74-110 mg/dL
ALT	160	0-41 IU/L
AST	153	5-40 IU/L
Alkaline phosphatase	513	40-129 IU/L
Total bilirubin	9.5	0-1.2 mg/dL
Calcium	9.4	8.6-10.3 mg/dL
Total protein	6.8	6.6-8.7 g/dL
Albumin	3.1	3.5-5.2 g/dL
Magnesium	2.0	1.6-2.6 mg/dL
Phosphorus	N/A	2.5-4.5 mg/dL
WBCs	9.4	4.5-11x10^9^/L
RBCs	2.62	4.5-5.9x10^12^/L
HGB	6.3	13.5-17.5 g/dL
HCT	20.4	41-53%
Platelet	467	150-400x10^9^/L

The case was discussed in a tumor board meeting to determine the best course of treatment, and the team agreed to use chemotherapy followed by chemoradiation as an adjunctive treatment. The patient was started on leucovorin calcium (folinic acid), fluorouracil, irinotecan hydrochloride, and oxaliplatin (FOLFIRINOX), but was lost to follow-up after completing two cycles. He was admitted 18 months later for a pulmonary embolism and was discharged on apixaban. CT of the abdomen and pelvis at that time showed multiple liver lesions and sub-centimeter nodules in the lungs concerning metastasis (Figure 1B). The patient was admitted a month later for severe abdominal pain and underwent an EGD for melena and hematemesis, which revealed a non-bleeding anastomotic ulcer and erythematous gastric mucosa. Liver biopsy showed metastatic pancreatic adenocarcinoma. Oncology was re-consulted, and the patient agreed to palliative chemotherapy and received seven cycles of FOLFIRINOX before being lost to follow-up.

## Discussion

Ampullary adenocarcinoma is a rare gastrointestinal malignancy that has been found to be slightly more common in males and with a wide age range at the time of diagnosis [2]. With the advanced endoscopic modalities over the past decade, the incidence of those tumors has increased. The presence of three main histological subtypes (intestinal, pancreaticobiliary, and mixed subtypes) has been established based on the histological pattern, each with distinct characteristics and prognostic implications. The pancreatobiliary subtype is the most common, accounting for 50-60% of cases. The intestinal subtype, associated with mutations in *APC* and *PI3KCA*, represents 25-35% of cases. The mixed subtype, exhibiting features of both the pancreatobiliary and intestinal subtypes, constitutes 10-20% of cases [5]. These statistics highlight the diversity of ampullary adenocarcinoma.

It remains incredibly challenging to distinguish primary ampullary carcinoma from periampullary malignancies preoperatively. The diagnosis of ampullary adenocarcinoma involves various diagnostic imaging techniques, endoscopic procedures, and histopathological evaluation. These studies include computed tomography (CT) scan, MRI, MRCP, and ERCP, with each modality playing a key role in determining the extent of disease and presence of metastasis. ERCP with biopsy is often performed to obtain histopathological confirmation but carries a risk of complications such as pancreatitis and bleeding [2]. This case report demonstrates the critical importance of precise pathological diagnosis. Correct histological classification of ampullary adenocarcinoma is crucial for its characterization since it has recurring prognostic implications.

In the intestinal subtype, the tumor exhibits glandular structures similar to normal intestinal epithelium, with well-formed glands lined by tall columnar cells showing brush border and goblet cell differentiation. The histological differentiation is supported by the presence of COX-2 in the intestinal subtype [1]. The intestinal subtype of ampullary adenocarcinoma is postulated to arise from premalignant precursor lesions and is commonly associated with K-RAS mutations [4]. Studies suggest that this subtype has a better prognosis compared to the pancreaticobiliary type, as patients with the intestinal subtype have a more favorable clinical outcome characterized by longer overall survival and better response to treatment (16 vs 115.5 months, respectively, P<0.001) [1,2].

In the pancreaticobiliary subtype, the tumor consists of infiltrating tumor cells with a substantial fibrous stroma and irregular glandular formations. These tumors are known to be aggressive, showing a higher likelihood of vascular invasion and lymph node metastasis, leading to a worse prognosis compared to the intestinal subtype. The mixed subtype of ampullary adenocarcinoma is characterized by having features of both the intestinal and pancreaticobiliary subtypes. It can exhibit a heterogeneous histological pattern with areas of well-formed glands resembling the intestinal subtype and areas of infiltrating tumor cells resembling pancreaticobiliary subtypes. The prognosis of mixed subtype is variable and depends on the proportion of each subtype present in the tumor. Immunostaining for CK7, CK20, and CDX2 can be beneficial in diagnosing ampullary adenocarcinoma by differentiating it from other malignancies such as pancreatic ductal adenocarcinoma, distal CBD carcinoma, and duodenal adenocarcinoma. Ampullary adenocarcinoma will typically show a CDX2+/CK7-/CK20+ staining pattern [1].

In early stages, periampullary carcinomas are treated with surgical resection, like other pancreatic carcinomas, via the Whipple procedure (pancreaticoduodenectomy), which is the gold standard [2]. The procedure's goal is to remove the tumor and surrounding lymph nodes as well as any affected tissue. This is accomplished by removing the head of the pancreas, the duodenum, the distal bile duct, and the gallbladder. The decision to perform the Whipple procedure depends on multiple factors, including the patient's overall health status, tumor stage, and location. The operation is suitable for those with tumors that can be removed surgically and patients who are suitable for undergoing the procedure. It is a major surgery with many potential risks and complications. It is possible to perform a curative surgery in approximately 50% of ampullary carcinomas, as compared to pancreatic adenocarcinoma, which carries less than 10% chance at curative surgery. Patients who have undergone surgical resection are generally offered adjuvant chemotherapy (ACT) with or without radiotherapy on a case-to-case basis to enhance the likelihood of recovery [2]. However, despite modern advances in cancer care and research, there remains a clear absence of evidence-based treatment standards for rare malignancies such as ampullary adenocarcinoma.

The systemic therapy for ampullary adenocarcinoma is used in all stages of the disease, including neoadjuvant therapy, adjuvant therapy, and first-line therapy for locally advanced, metastatic, and recurrent disease. The choice of systemic therapy is often pulled from data and clinical experience in the setting of pancreatic cancer, colorectal cancer, and biliary tract cancer. For the pancreatobiliary/mixed subtype, recommendations are typically derived from guidelines for pancreatic or biliary tract cancer. For the intestinal subtype, recommendations are drawn from the National Comprehensive Cancer Network (NCCN) Guidelines for Colon Cancer, which frequently suggest a 5-FU-based regimen such as FOLFOX (a combination of folinic acid [leucovorin], fluorouracil [5-FU], and oxaliplatin) [6]. For the pancreaticobiliary subtype, clinicians often refer to the NCCN guidelines for pancreatic cancer or hepatobiliary cancers, which often recommend a gemcitabine-based regimen [6]. The potential utility of many regimens in individual patients must be carefully evaluated by the treating physicians based on interpretation of data and drug risk/benefit profile [5].

In a retrospective study, Nassour et al. reported that the use of adjuvant therapy was associated with improved survival compared to just surgical management, with more significant outcomes in patients with more advanced disease [7]. Out of the 4,190 patients they studied, 63% were simply observed post-surgical resection, and 21% and 16% received ACT and adjuvant chemoradiotherapy (ACRT), respectively. Patients who received ACT were found to have a median overall survival of 47.2 months compared to 35.5 months in the control group (HR: 0.82; 95% CI = 0.71-0.95). Similarly, patients who received ACRT had a better survival rate of 38.1 months versus 31.0 months when compared to the control group (HR: 0.84; 95% CI = 0.72-0.98) [2].

The prognosis can vary depending on the tumor stage. Patients with tumors in the early stages, such as stage I or II, that can be subjected to complete surgical resection have better survival rates compared to those with more advanced tumors. High-grade tumors or tumors in advanced stages, such as stage III or IV, are associated with worse outcomes due to a higher likelihood of recurrence and metastasis. The prognosis of ampullary adenocarcinoma depends on various factors, including tumor stage, subtype, and age of the patient. Early detection and surgical resection of tumors are the mainstay of treatment. Resected tumors have five-year reported survival rates that range from 30% to 60%. Timely diagnosis and management in patients diagnosed with ampullary adenocarcinoma is necessary to lead to favorable outcomes [1].

## Conclusions

While ampullary adenocarcinoma is a rare entity, its prevalence has increased over the past decade due to advanced endoscopic and imaging modalities, leading to increased detection. In addition to imaging needed to determine the extent of this disease, biopsy is imperative to establish the specific type of ampullary tumor that has distinct characteristics and prognostic implications. Our case also highlights the risk of recurrence and metastasis following surgical excision via the Whipple procedure. Given its rarity, there remains a need for further research to establish evidence-based standard of care treatment paradigm.
